# Innovative Hurdle Technologies for the Preservation of Functional Fruit Juices

**DOI:** 10.3390/foods9060699

**Published:** 2020-06-01

**Authors:** Predrag Putnik, Branimir Pavlić, Branislav Šojić, Sandra Zavadlav, Irena Žuntar, Leona Kao, Dora Kitonić, Danijela Bursać Kovačević

**Affiliations:** 1 Faculty of Food Technology and Biotechnology, University of Zagreb, Pierottijeva 6, 10000 Zagreb, Croatia; lkao@pbf.hr (L.K.); kitonicdora@gmail.com (D.K.); 2 Faculty of Technology Novi Sad, University of Novi Sad, Bulevar cara Lazara 1, 21000 Novi Sad, Serbia; bpavlic@uns.ac.rs (B.P.); sojic@tf.uns.ac.rs (B.Š.); 3 Department of Food Technology, Karlovac University of Applied Sciences, Trg J. J. Strossmayera 9, 47000 Karlovac, Croatia; sandra.zavadlav@vuka.hr; 4 Faculty of Pharmacy and Biochemistry, University of Zagreb, Ante Kovačića 1, 10000 Zagreb, Croatia; izuntar@pharma.hr

**Keywords:** functional fruit juice, hurdle technology, non-thermal processing, preservation, quality, probiotic

## Abstract

Functional nutrition, which includes the consumption of fruit juices, has become the field of interest for those seeking a healthy lifestyle. Functional nutrition is also of great interest to the food industry, with the aims of improving human health and providing economic prosperity in a sustainable manner. The functional food sector is the most profitable part of the food industry, with a fast-growing market resulting from new sociodemographic trends (e.g., longer life expectancy, higher standard of living, better health care), which often includes sustainable concepts of food production. Therefore, the demand for hurdle technology in the food industry is growing, along with the consumption of minimally processed foods, not only because this approach inactivates microorganisms in food, but because it can also prolong the shelf life of food products. To preserve food products such as fruit juices, the hurdle technology approach often uses non-thermal methods as alternatives to pasteurization, which can cause a decrease in the nutritional value and quality of the food. Non-thermal technologies are often combined with different hurdles, such as antimicrobial additives, thermal treatment, and ultraviolet or pulsed light, to achieve synergistic effects and overall quality improvements in (functional) juices. Hence, hurdle technology could be a promising approach for the preservation of fruit juices due to its efficiency and low impact on juice quality and characteristics, although all processing parameters still require optimization.

## 1. Introduction

Functional foods are products with added active substances, which if consumed in adequate quantities not only provide basic nutrition, but also have a positive impact on general health or contribute to reduction of the risks for developing certain diseases [1]. Consumers are now increasingly looking for products that are safe and natural, have Generally Recognized as Safe (GRAS) status, and are produced using sustainable or ecological technology [2]. By 2020, it is estimated that the global market for functional foods will reach $304.5 billion, with an average annual growth of 8.5%, despite the fact that the development of new (functional) foods is a fairly costly and complex business [3]. In light of these trends, consumers have a tendency to purchase fruit juices or beverages in order to satisfy their daily nutrient needs and improve their wellbeing in a quick and easy way. According to the National Health Service (UK), the daily recommendation of 5 servings of fruits and vegetables (“five a day”) can be adequately substituted with 150 mL of 100% fruit or vegetables juices [4]. It has been demonstrated that biological active compounds (BACs) in the organism are much more easily absorbed and digested if they are ingested from juices than those from whole plant tissue.

Hurdle technology is a set of methods used for inactivation of microorganisms in food preservation. Hurdles are food preservation factors, which are combined to achieve certain food quality and stability, in terms of the temperature, pH, redox potential, water activity, preservatives, and competitive microorganisms. Today, more than sixty hurdles are known to have an effect on food stability and quality, as well as microbial stability [5]. Not all hurdles are combined together, nor are they all used to preserve one food product. The effect that a hurdle has on the food depends on the intensity, which affects the microbial stability and means the intensity needs to increase, while if the hurdle affects the food quality the intensity needs to decrease. Classical heat treatment (pasteurization) of fruit juices disrupts the stability of the thermolabile BAC molecules, so the application of non-thermal technologies using the “hurdle concept” could be a promising solution in order to meet the consumer criteria of quality, nutritional value, and product safety.

To preserve food using hurdle technology, it is necessary to create a hostile environment for microorganisms. This environment can either cause the death of microorganisms or slow down their growth, largely depending on how microorganisms react to the treatment. These reactions are being researched as methods of food protection, together with the effects of hurdle technology on homeostasis, stress reactions, metabolic exhaustion of microorganisms, and multitarget preservation, all of which are essential for food safety.

The use of only a single technology may cause damage to microorganisms that they may recover from during food storage. However, if this recovery is effectively prevented by using a combination of additional hurdles, the microbial cells will not be able to grow and higher inactivation levels could be reached. In this respect, most of the current research studies are focused on high-power ultrasound (HPU), pulsed electric field (PEF), and high-pressure processing (HPP) [6]. These technologies have low greenhouse gas emissions and energy consumption and reduced environmental impacts; hence, they are considered as sustainable technologies that are in line with Agenda 2030, which is geared towards their use [7]. HPP represents a technology with a physical effect as the main preservation factor; thus, it is often used as a hurdle technology. When processing fruit juices, PEF has been applied as an electrotechnology within hurdle technology in combination with numerous other options, e.g., antimicrobial additives, thermal treatment, ultraviolet light (UV), and manothermosonication (MTS). Similarly to HPU, it is also commonly combined with numerous other technologies, e.g., UV-C light, pulsed light, and high pressure. [8]. However, interestingly the use of HPU and PEF as a combination hurdle technology has not been sufficiently explored in terms of the ecological and economical potential as a substitution for pasteurization in fruit juices. This is despite previous research results implicating these two non-thermal techniques as having synergistic potential for overall quality improvements of (functional) juices.

## 2. Functional Fruit Juices for Delivery of Bioactive Compounds, Probiotics, and Prebiotics

In today’s world, there is a growing trend toward health and fitness. Accordingly, the food industry has directed their efforts towards producing healthier and more nutritious products that will satisfy consumer demands. One way to go about this is to design completely new products, while another way is to add functional ingredients to already existing products. Functional ingredients are substances that are beneficial for our health, e.g., carotenoids, phenolic acids, flavonoids, polyunsaturated fatty acids, probiotics, minerals, and vitamins. Because of the fast-paced Western lifestyle, people have less time to eat fresh fruit and prefer to consume fruit juice instead. Hence, juice represents an excellent vehicle for the delivery of bioactive ingredients.

Some fruit juices are already highly valued because of their unique nutritive composition. However, new products in the fruit sector are constantly being designed for several reasons. People are willing to purchase healthier products, such as those that are organically made, those they find appealing, and new and different fruit juices. The demand for functional fruit juices is increasing rapidly. Superfruits with high levels of bioactive compounds, such as goji berries, areola, noni, and acai, are already added to some fruit juices, as well as prebiotics, probiotics, omega-3 fatty acids, botanicals, and isoflavones from soy, in order to make products functional [9,10]. In 2019, one study tried to identify interactions between bioactive compounds and the production of functional fruit juice. Cranberry bush juice was added to pear juice in order to make it functional. Finally, juice made from 95% pear and 5% cranberry bush (PC2) and juice made from 97.5% pear and 2.5% cranberry bush (PC1) were the most popular products among consumers. These juices also had good total soluble solid/total titratable acidity (TSS/TTA) ratios, and for the PC2 juice the interactions between bioactive compounds were the highest [11].

The economic value of juice production was confirmed over the last 5 years with statistical data from the European Union (EU), where production of juices not made from concentrate increased by 5.4%, while production of freshly squeezed juices grew by 4.8%. Simultaneously, the production of nectars and other types of fruit beverages declined by 3.8% and 1.6%, respectively [12]. In this context, domestic autochthonous fruit varieties are attractive raw materials used for the production of functional fruit juices that provide profitable food products on the market, while preserving traditional agriculture, contributing to the strengthening of local economies, and consequently contributing to the welfare of society.

Fruits and their juices contain significant amounts of different BACs, which individually or mutually have a significant impact on improving human health. The most common types of BACs in the majority of fruit juices are polyphenols, which are antioxidants with anti-inflammatory activities. Polyphenols bind free radicals, participate in the inhibition of pro-oxidative processes, and significantly influence chronic inflammation or lessen the burden of various medical pathologies, such as cardiovascular diseases, forms of cancers [13,14,15,16,17], and lipid oxidation in plasma [16,18]. There is a large body of evidence showing that polyphenols are true aids to human health and wellbeing; however, there is controversy associated with their utilization in food production. In order to obtain true functional product with medicinal value, it has to be designed according to strict rules and assessed with randomized, double-blind, placebo-controlled clinical trials [19].

In addition to the usual nutritional value, in order to provide functionality and added value to fruit juices there is the possibility of adding prebiotics and probiotics, which have been examined recently. The term “probiotic” refers to mixtures made of one or more bacterial species that act positively on the health of the host to which they are introduced by improving the properties of the hosts intestinal microflora [20,21,22]. The most common beneficial effects of probiotics are the reduction of lactose intolerance; reduction of cholesterol levels; stimulation of the immune system; and increased absorption of minerals with antimicrobial, anticarcinogenic. and antihypertensive effects [23]. Probiotic bacteria are traditionally added to fermented dairy products; however, their applicability is limited by lactose intolerance or by diets that require cholesterol restriction [23]. One solution is fruit juice, which stands out as a new category of carriers for probiotic bacteria [24,25,26,27] and has the advantage of containing suitable nutrient contents for their growth, hence stabilizing the probiotic product [23]. The use of probiotics improves the nutritional properties of the product, while in a fruit juice the addition of probiotics enhances the antioxidant activity (limited to certain strains of lactic acid bacteria) [23]. The most commonly used probiotic bacteria in fruit preparations are the different strains of *Lactobacillus plantarum*, *Lactobacillus acidophilus*, *Lactobacillus helveticus*, *Lactobacillus casei*, *Lactobacillus paracasei*, *Lactobacillus rhamnosus*, *Lactobacillus reuteri*, *Bifidobacterium breve*, *Lactobacillus gasseri*, *Lactobacillus crispatus*, and others [28]. Selection of the right bacterial strains for the production of fruit preparations is crucial, as ensuring their stability, survival, and functionality is more challenging than in fermented dairy products [23,29]. Despite all the positive attributes that probiotics can bring to human wellbeing, there are number of challenges that are associated with their consumption and industrial application.

## 3. The Use of Non-Thermal Technologies within the Hurdle Concept for the Preservation of Functional Fruit Juice

Currently, consumers demand that fruit juices are minimally processed, contain no additives, and have an extended shelf life. Recent studies have confirmed that consumers are often ready to pay a higher price for premium products of high sensory quality; therefore, there is currently high motivation to develop and produce premium quality juices. Such fruit juices are produced directly by squeezing or cold-pressing and are not subjected to any further processing—they are simply stored at a temperature of 2–5 °C, meaning they last for only a few days (1–3 days). On the other hand, the results of a major study involving over 160 US and EU fruit juice producers stated that microbiological safety is a key factor in product protection within a market. As many as 92% of manufacturers have encountered subsequent contamination (yeast or mold) in their products [30]. Functional fruit juices are short-term products, so classical (thermal) pasteurization (70–121 °C/30–120 s) assures they have an adequate shelf life, even though this treatment reduces the nutritional value [31,32] and the quality of the juice [33]. To that end, a recent study aimed to discover how physical, chemical, and sensory properties of tropical fruits juices change after pasteurization (85 °C, 30 s) and during 90 and 180 days of storage [34]. In the aforementioned study, the authors used two formulations made from different ratios of acerola cherry (*Malpighia emarginata* D.C.), cashew apple (*Anacardium occidentale* L.), yellow mombin (*Spondias mombin*), pineapple (*Ananas comosus* L.), acai (*Euterpe oleracea*), and camu-camu (*Myrciaria dubia*) fruit. The results revealed that the pasteurization did not affect the total polyphenolic content or antioxidant activity of either formulation but caused a 7% reduction of ascorbic acid. A higher carotenoid content in the pasteurized samples as compared to the control sample was explained by the combination of homogenization and heat treatment, which caused disruptions of cell membranes and the protein–carotenoid complex, making carotenoids more accessible for extraction.

Non-thermal processing technologies are able to extend the shelf life while retaining the nutritional and sensory value. These technologies operate at lower temperatures and with shorter processing times, while assuring microbiological safety, inactivation of the enzymes, and higher stability of the BACs in the juice. Recently, for the purposes of extending the shelf life and retaining the quality of juices, combinations of thermal and non-thermal technologies were tested as “hurdle” technologies. Here, these technologies are applied in a certain sequence, so that each of the non-invasive (“milder”) processing conditions gives the best results in terms of extending the shelf life and sensory quality of fruit juices [35,36]. In this respect, most of the research studies today are HPU, PEF, cold plasma (CP), high-voltage electrical discharge (HVED), and HPP technologies [6]. The specificity of these concepts is reflected in the synergistic effects of various inhibition mechanisms or by the inactivation of target microorganisms [37]. Therefore, the innovative “obstacle” technology is an advanced approach to fruit juice processing with the potential to meet the high demands of consumers and manufacturers [30].

In recent decades, there has been an extensive increase in the number of commercially available foods processed using non-thermal technologies. For instance, food products processed using non-thermal technologies such as HPP have been marketed in Japan since 1990 and in the United States and Europe since 1996 [38]. At the same time, consumers do not have clear knowledge and information about the new technological processes applied to food; therefore, their attitudes are often negative towards such processing. This is the reason why consumers often consider processed food unhealthy and are often skeptical about its quality [39]. A recent study revealed that Brazilian consumer perception of food technologies was influenced by the degree of food technology neophobia [40]. Fresh, cold-pressed, and non-pressurized juice were mostly associated with healthy and natural products, whereas pasteurized juice and pressurized juice were associated with processed products and unhealthiness.

### 3.1. Pulsed Electric Field Processing

The application of pulsed electric field processing technology as an alternative to pasteurization of fruit juices has shown good results, without significant deterioration of nutritional or sensory juice quality [41,42]. PEF processing involves the application of a high voltage (about 50 kV cm^−1^) in a very short time (μs to ms) to food placed between two electrodes. Here, the main parameters are temperature and time, electric field strength, and energy input [30].

Microbial inactivation by PEF is based on the rupture of microbial cells due to differences in the potential outside vs. inside the cells [43]. When the difference in these potentials reaches a critical value characteristic for each microorganism, electroporation occurs, inducing irreversible structural changes and inactivation of microorganisms [44]. Electroporation can be reversible, meaning that the intensity of the treatment may not inflict sufficient damage to the cells or that the pores on the cellular membrane might be too small. This indicates that microbes can recover from partial inactivation, so PEF is frequently combined with different hurdles for full safety.

The efficiency of PEF depends on the experimental conditions, food composition, and electrical conductivity of the food. If the conductivity is higher than 6 μs, the food is harder to process with this technology [43]. Additionally, PEF depends on several factors, such as the electrical field strength; processing time; width, frequency, and shape of the pulse; polarity; energy; and applied temperature [43]. Studies have indicated that microbial characteristics (e.g., cell size and shape, type of cell envelope) alone seem not to have the expected impact on PEF resistance [45]. Furthermore, PEF could not stand alone as an efficient treatment for inactivation of spores under mild processing conditions, meaning it should be combined with other preservation techniques to achieve the required results. Moreover, the other technological limitations of PEF, such as the high initial cost of equipment, still do not allow the complete commercialization of food products processed using PEF.

Liquid foods without bubbles are the most suitable for PEF treatment due to the presence of charged molecules that can transfer electric charges formed during treatment [42]. This means that PEF can also be used for processing of dairy and alcoholic beverages. Several studies have confirmed the lower efficacy of PEF in treatment of juices containing higher amounts of macronutrients (e.g., fats and proteins) in comparison to simple microbial suspensions [46]. With respect to microbial inactivation, physicochemical juice parameters such as pH, water activity, and soluble solids are some of the main factors that can affect the PEF treatment. Table 1 gives an overview of the application of PEF for the preservation of functional fruit juices and beverages over the last 5 years.

Many studies have confirmed the superior polyphenolic, mineral, and free amino acid contents, as well as better preservation of native color in PEF-treated juices as compared to thermally treated juices. High-intensity PEF (HIPEF) was able to improve the bioaccessibility of bioactive antioxidants in several functional beverages [47,48]. Recent experiments showed that PEF preprocessing applied to whole raw tomatoes (*Lycopersicum esculentum* cv. Raf) increased concentrations of individual carotenoids (phytofluene, phytoene, lycopene, δ-carotene, lutein, γ-carotene, β-carotene) [49]. In particular, the concentrations of phytoene and phytofluene were increased by 178% and 131%, respectively, in tomato products treated with PEF and compared to untreated fruit. The authors explained this as being caused by activation of the secondary metabolism in the treated tomatoes as a strategy to overcome unfavorable stress conditions. Moreover, the authors revealed that PEF treatment applied to tomato fruit increased the bioaccessibility of δ-carotene (2%), β-carotene (53%), lutein (125%), lycopene (137%), and γ-carotene (527%). These results strongly confirmed that PEF could lead to an easier release of carotenoids from the tomato matrix [49]. On the other hand, PEF had a negative effect on the cellular membranes of tomatoes that led to softening of the vegetable [50]. In conclusion, PEF was implicated as a beneficial preparation technology for production of tomato-based products with high carotenoid concentration.

As mentioned earlier, PEF can inactivate microorganisms in juices while retaining quality (e.g., color, soluble solids, viscosity, content of bioactive compounds, etc.), which makes it an advantageous method for preserving foods over an extended shelf life [53,58]. The effects of PEF on the sensorial and nutritional quality of mango juice right after treatment and during storage (75 days) were recently tested [59]. Experiments showed that there were no significant changes in conductivity or in the pH of the samples after treatment. However, pH did decrease slightly during storage for 59 days. Furthermore, the Commission Internationale de l’Éclairage LAB (CIELab) lightness (L*) remained the same during storage, which indicated good preservation of color. The native yellow color of mango juice remained unchanged, as PEF inactivates polyphenoloxidase (PPO) and peroxidase (POD), which catalyze the browning reactions. Electrotechnologies may induce structural and conformational changes in food enzymes, which could limit their activity, thus strongly affecting product quality.

Another study aimed to evaluate and compare the effects of processing via high-intensity pulsed electric fields (HIPEF) vs. thermal pasteurization (90 °C/60 s) on the nutritive and physicochemical parameters of date juice (variety Bou-Hattem) before and after storage for 5 weeks at 4 °C. In comparison to pasteurization, HIPEF better preserved the physicochemical characteristics and even caused an increase in the concentrations of some bioactive compounds in fruit juices. Moreover, HIPEF-treated date juices, in comparison to untreated and thermally processed samples, retained quality better during storage. A possible reason could be the improved extraction of intracellular contents due to permeabilization of plant cells, which is enhanced by PEF [60]. As a result of food processing and storage, some biochemical reactions may occur, which could promote the formation of new compounds. Those new compounds are usually formed by constituents (e.g., polyphenols) that are stored in polymers, such as pectin or cellulose, and could be released during processing or storage [61].

Non-enzymatic browning reactions were slowed down by PEF, which resulted in a low concentration of hydroxymethylfurfural (HMF) in treated date juice. HMF, as an indicator of non-enzymic browning, is probably a result of Maillard reaction in the presence of a reducing sugars (e.g., glucose and fructose) and acids under high temperature [62]. The slow browning rates observed in HIPEF juices in comparison to pasteurized ones could be related to the high retention of acids in date juice [61]. The reason for this could be the preservation of ascorbic acid oxidation in HIPEF. Non-oxidized ascorbic acid cannot provide reactive carbonyl groups, which are precursors of non-enzymatic browning that lead to the formation of HMF.

Another study on Pinot noir grape juice was done to evaluate exposure to PEF and the effects this has on the quality and nutritive value (e.g., contents of anthocyanins, total phenolics, and vitamin C). Grapes have a number of anthocyanins, phenolic acids, flavonols, flavan-3-ols, and stilbenes. Prolonged maceration is commonly used to increase the concentrations of these compounds, which are released from grape skins, while PEF can be used to accelerate this process. To that end, after preparation the grape juice was treated with PEF (constant pulse width of 20 µs; pulse frequency of 50 Hz; electric field strength of 1.5 kV cm^−1^; “PEF Low” pulse numbers of 243, and “PEF High” of 1033) and subsequently macerated. The grape juice treated with PEF had better yield during the first four days of maceration than untreated juice. Enhancement of juice yield and improvement of mass transport of juice from the grape pulp is credited to the PEF permeabilization of cell membranes. Juices that were processed with “PEF low” had increased vitamin C content during the first two days of maceration. However, when the maceration was longer than 8 days, PEF-treated juices exhibited higher degradation of vitamin C than non-treated juices. It seems that the higher release of vitamin C in early maceration increased the available total vitamin C for oxidation due to the higher susceptibility to oxidation and degradation with prolonged maceration. “PEF high” samples showed an increase of total phenols after two days of maceration. “PEF low” and “PEF high” juices had higher contents of malvidin-3-*O*-glucoside (anthocyanin) after eight days of maceration as compared to control samples, suggesting that PEF has the potential to maximize the release of phenolics much more effectively than conventional maceration alone. In addition, the maceration process was successfully shortened from 14 to 8 days, thus confirming the potential of PEF in grape juice processing [30].

### 3.2. High-Power Ultrasound Processing

The use of ultrasound (US) as an alternative to pasteurization in fruit juices has great potential, as the acoustic energy is immediately transmitted through the entire volume of juice, which significantly shortens the treatment time. In addition, its lower energy consumption and potentially lower costs makes this method appropriate for industrial usage [63]. Fruit juice processing commonly combines high-power ultrasound processing with high-intensity ultrasonic waves (10–1000 Wcm^−2^) at low frequencies (20–100 kHz). Due to the high power, ultrasonic waves in juice cause physiochemical changes, accelerating biochemical reactions and causing enzymatic and microbial inactivation [64]. Expansion vortexes are formed due to the passage of the ultrasonic waves through the liquid, forming small bubbles and negative pressures, which is defined as the cavitation phenomenon [65]. The collapse of the cavitational bubbles inside and around microbial cells causes the pressure gradient, which induces mechanical damage to the cells. Additionally, free radicals that damage the cell walls are formed during HPU treatment, with spontaneous formation of bactericidal hydrogen peroxide further enhancing the microbial inactivation [64]. Studies have documented that ultrasound processing has the potential to achieve a 5-log reduction in foodborne pathogens in fruit juices. Although ultrasonic waves cannot guarantee bacterial spore inactivation, synergic effects with other technologies (e.g., in hurdle technology) could enhance the effectiveness of spore-forming bacteria inactivation [66,67]. The main disadvantages found during ultrasound processing are related to process scale-up and quality changes in the product. Parameters influencing the effectiveness of HPU treatment in fruit juice processing are the ultrasonic strength, frequency, ultrasonic probe diameter, amplitude, time of treatment, and temperature [64]. This technology makes it possible to obtain microbiologically stable fruit juice with preserved BACs and improved bioavailability [68,69]. Table 2 provides a summary of the application of HPU for the preservation of functional fruit juices and beverages over the last 5 years.

HPU can provoke microstructure changes of treated juice, particularly the rupturing of cellular walls. More precisely, the physical force of ultrasound causes the dispersion of the intracellular components into the juice, causing higher injury to the cellular structures, which occurred through elongating the HPU processing time [70]. These structural changes may greatly contribute to the improved physical properties of juice with respect to rheological properties, color attributes, cloudiness, and water-soluble pectin.

The addition of various prebiotic carbohydrates in orange juice, such as xylooligosaccharides (XOS), has been introduced through high-intensity ultrasound (HIUS) for the engineering of functional beverages with similar sensory characteristics to those of fresh products [71]. The quality parameters and XOS stability of functional beverages were investigated under the effects of nominal US power values (W) of 0 (control/untreated sample), 300, 600, 900, and 1200 W over 10 min. HIUS treatment over a temperature range of 51–88 °C did not affect the chemical stability of the XOS. Therefore, XOS-enriched beverages preserved the functional properties of added prebiotics. However, the negative effects of HIUS were noticed on the stability of ascorbic, malic, and citric acid contents, as well as on the total phenolic content and antioxidant activity.

HPU treatment may prevent the activity of browning enzymes such as PPO and POD, maintain the taste and nutritional value, and the improve stability of juice with respect to conventional heat treatment [83]. The efficiency of sonication depends on the intensity of treatment. Accordingly, it can cause inactivation of microorganisms, increase the concentration of bioactive compounds, or accelerate fermentation. The impact that ultrasound has on a juice depends on the juice itself. A recently conducted experiment on strawberry juice showed that ultrasound did not affect the color of the juice, nor did it form “off flavors”. Moreover, it increased the acidity and sweetness of the juice and the scavenging activity (i.e., antioxidative capacity) of the samples by 75%, which continuously grew during storage. After the treatment, the concentration of phenolic compounds was higher than in fresh juice, likely caused by cavitation or addition of hydroxyl radicals to the phenolic rings during the treatment.

The positive aspects of ultrasound treatment were documented in a study on the effects of HPU on passionfruit juice. The juice was treated with a frequency of 20 kHz for 10 min, after which it was stored with untreated samples at 4 and 10 °C for 10 days. Samples were taken every other day and their color, pH, ascorbic acid content, and microbial count were measured. Results of this experiment showed that HPU did not cause sufficient cell damage to start immediate microbial inactivation. However, sonication treatment successfully reduced the aerobic mesophilic bacteria and yeast counts. Due to HPU treatment, the color parameters lightness (L*), a* (redness), and yellowness (b*) of passionfruit juice changed and the color was lighter and greener. The degradation of ascorbic acid was significant, but was lower than the decrease caused by pasteurization, hence making the HPU treatment a better method for preservation of fruit juices. Further studies showed that the juice was stable for 10 days after sonication because there were no significant changes in microbial quality of the product, as opposed to non-sonicated samples [84]. Aside from juices, the positive impacts of ultrasound extend to the quality of meat, fish, cheese, chocolate, vegetables, emulsions, and other products. Although ultrasound is commonly used as a replacement for pasteurization, it can also be applied as a pretreatment for foods that are processed by conventional methods, such as freezing, defrosting, drying, extrusion, slicing, and frying.

Non-clarified strawberry juice was exposed to HPU and thermal treatment (TT) in order to compare their influences of the quality and nutritional value during 10 days of storage. The evaluated properties were color, total soluble solids, total acidity, microbial growth, total phenolic content, and antioxidant activity (DPPH assay). The juice was sonicated at 40 kHz for 10 and 30 min at 90 °C for 60 s. All HPU samples showed a constant color right after the treatments, which started to degrade during storage (the largest drop was observed on the third day). TT samples had higher color changes after the treatment compared with untreated samples, but after the initial change the color remained stable over the storage period. All samples showed no “off odors”, but HPU samples were sweeter than TT juice, probably due to the breakage of cellular structures that contained sugars. HPU and TT samples had similar total soluble solid contents, total acidity, and nutritive value, while TT inhibited microbial growth better than HPU [85].

Conversely, the negative side effects of ultrasound on juice quality can include changes of color, viscosity, formation of free radicals and off flavors, as well as degradation of lipids. In some juices, sonication reduced the amount of ascorbic acid, anthocyanins, and carotenoids. These factors, as well as the cost of ultrasound treatment, affect (and limit) the application of ultrasound in the food industry [86].

Due to the low lethality of ultrasound, a number of microorganisms, especially bacteria spores, can survive HPU treatment. To insure a higher lethality, HPU is often combined with pressure, heat, or both at the same time. These combinations are more energy-efficient than ultrasound alone, but cannot be used for preservation of thermosensitive products. For example, thermosonication can accelerate enzymatic inactivation and sterilization by shortening the treatment time and decreasing the intensity of sterilization and the corresponding negative side effects [87]. Moreover, the effectiveness of ultrasound can be improved when it is combined with chlorination, extremes of pH, and addition of preservatives [88].

### 3.3. High-Pressure Processing

High-pressure processing is considered the best non-thermal method used for food preservation. Food products that can be processed with HPP are low- to medium-moisture semisolids and solids (vacuum packed), high-moisture solids (vacuum packed), and high-moisture liquid food (bottled). Products are packed before the process, which decreases the chance of postcontamination. After this, products are placed in baskets and lowered into a vessel. The vessel is then filled with water and the products are treated with high hydrostatic pressure, which can be as high as 800 MPa. The pressure is evenly spread throughout the vessel, which assures that the product will be properly processed [89]. By applying HPP, non-covalent bonds in enzymes, ribosomes, and molecules of the cell membranes are ruptured, resulting in microbial inactivation. Since bacterial spores are quite resistant to HPP, they are often combined with high temperature to make the process more efficient [86]. The efficiency of HPP depends on the pressure, temperature, time, and pH [86].

Governmental authorities in the USA, Europe, and Canada have approved the use of HPP for reducing pathogens in juices and extending their shelf life. With an annual growth of at least 1%, fruit beverages show the fastest growth in the HPP food market, so such products may hold a higher market share than meat products that utilize HPP technology. However, the main technological limitations of HHP are the high investment costs for the equipment and the batch operation mode. The application of HPP for the preservation of functional fruit juices and beverages over the last 5 years is outlined in Table 3.

When HPP (190 MPa, 60 °C) was compared with pasteurization (90 °C, 5 min), reduced numbers of bacteria and yeast were shown in mango juice, and the shelf life of the juice was prolonged for up to 60 days while stored at 4 °C. Furthermore, losses of ascorbic acid, carotenoids, and total phenols were reduced as compared to control samples [86]. HPP has good selectivity for destruction of pathogenic bacteria while retaining good probiotic bacteria. For instance, after HPP treatment, *Lactobacillus rhamnoses* GG probiotic bacteria were preserved. Some studies even show that after 30 days of storage, the bacteria had a content of over 108 CFU/mL, with a well-preserved nutritive profile. Hence, it is not surprising that consumers showed tendencies toward purchasing HPP-treated probiotic products rather than thermally treated products [90].

The inactivation of quality degradation by endogenous enzymes in fruit juices is needed to obtain a high-quality product. The effects of HPP on the inactivation of various enzymes in fruit juices, e.g., PPO, POD, pectinmethylesterase (PME), β-glucosidase, polygalacturonase, lipoxygenase, amylase, and hydroperoxide lyase, have been extensively studied [91]. One recent study aimed to compare white grape juice samples treated with HPP and TT with respect to microbial levels (aerobic bacteria, coliform bacteria, and yeast/mold), physiochemical and antioxidant properties, enzymatic activities (PPO and POD), and sensory analysis during storage. After 20 days of storage, enzymatic activity decreased by 50% in HPP samples, with significant drops in microbial levels. Moreover, HPP juice received a similar sensory evaluation as the fresh juice, while TT the reduced sensory appeal in the samples.

Bioaccessibility is another important nutritive parameter for evaluation of functional food products as fruit juices. A recent study confirmed that the bioaccessibility of phenolics in fruit juice may be enhanced by modulation of the process parameters and the composition of the food matrix. A comparison of the high-pressure homogenization (HPHP; 250 MPa) and T80 (80 °C/30 min) and T90 (90 °C/30 s) thermal treatments with the addition of milk was associated with phenolic bioaccessibility and activity in kiwi and pomelo juices. Both HPP and TT increased the contents of phenols in both juices, although the T80 sample was an exception because of the phenolic degradation in pomelo juice. Unfortunately, the bioaccessibility of free and total phenols did not increase as a result of the applied treatments. Moreover, in some samples the total phenolic content even decreased, while the addition of milk improved the bioaccessibility of free and total phenols. Here, the exception was soy milk, where the number of total phenols decreased. In kiwi juice samples, the best bioaccessibility was with the addition of semiskimmed milk, while in pomelo juice this resulted from the addition of the whole milk. The authors explained that the improvements were due to the fact that some phenols can create bonds with milk proteins, while others can create bonds with lipids. To conclude, under the tested circumstances the kiwi juice had better phenolic activity and bioaccessibility than pomelo juice [99].

Green asparagus juice was tested in terms of its microbial contamination, physiochemical properties, antioxidant activity, and changes in BAC content in HPP vs. TT treatments (121 °C, 3 min) and control (no treatment). The researchers used HPP at 200, 400, and 600 MPa for 10 and 20 min. HPP at 400 and 600 MPa reduced the number of microbes (total mesophilic bacteria) to the same values as the TT. Moreover, the physiochemical properties and pH did not change during the HPP treatment, which had more BACs (ascorbic acid, rutin, and total phenolic content) in comparison to the TT [100].

Apple juice samples (elephant apple) were treated with HPP (600 MPa/5 min) and TT (80 °C/60 s) for extended storage [101]. Samples were compared after treatment regarding quality parameters (pH, °Brix, total acidity, viscosity, color, and sensory analysis), nutritive value (antioxidant activity, total phenols, total flavonoids), and microbial load. Juice samples were analyzed over 60 days, during which pH, °Brix, and total acidity were similar among HPP and TT samples. However, color, antioxidant activity, total phenols, and total flavonoids were better preserved with HPP samples than TT samples. HPP samples had a lower microbial load than other samples. In conclusion, HPP was identified as a good technology for preservation of apple juice.

Cloudy apple juice was treated with HPP (600 MPa for 3 min) and TT (85 °C for 5 min) to examine changes in the quality and nutritive value. TT caused significant color changes, with an increase in L* and decreases in b* and a* CIELab values. Sugars and acids in the samples remained similar after both treatments. TT-treated juices showed higher amounts of aldehydes, ketones, alkanes, alcohols, and organosulfur compounds than HPP and untreated samples did. These likely resulted from Maillard and oxidative reactions, which are propagated with heating. The results showed that better retention of natural juice color was possible in HPP samples, while TT samples showed better inactivation of PPO and POD [102].

The advantages of using HPP in the food industry, aside from food conservation, include improved antioxidant properties in food components. For example, in strawberry juice, when both treatments HPP and TT are combined, this will decrease the total monomeric anthocyanin (TMA) and vitamin C contents. However, results showed that the TMA and vitamin C contents did not decrease with HPP but did during TT [103]. Another interesting nutritive benefit of HPP is the decrease of the glycemic index (GI). HHP processing of fresh mango puree may provide additional benefits for glycemic control, as it was shown that the mean GI for HHP-processed mango puree was significantly lower (32.7) in comparison with unprocessed puree (42.7) [104]. HPP can also increase the concentrations of amino acids, such as y-aminobutrimetric acid (GABA), which is created from glutaminic acid. To that end, brown rice was treated with HPP of 200 MPa. After 4 days of storage, the concentrations of all amino acids, including GABA, rose more significantly in HPP samples than in control samples [105]. HHP has been used as an alternative technology to improve the functional properties of various kinds of proteins. Bovine lactoferrin was treated by HPP at different pH values (4.0 and 6.0), at 300–700 ± 10 MPa for 30 and 60 min at 25 °C. Here, higher pressure induced changes in the tertiary structure of lactoferrin at pH 4.0, indicating its partial denaturation and aggregation. After being treated by HHP at pH 6.0, insoluble protein aggregates modified secondary structure. Therefore, in milk-based products, replacing pasteurization with HPP can result in higher solubility, foaming, and emulsifying properties in lactoferrins.

The HPP, HPU, and PEF samples were tested for their ability to reduce the power of the microbial load of *Escherischia coli* in strawberry juice. Samples were treated with HPP at 200–400 MPa for 2 min; HPU with 120 μm, 24 kHz at T = 25–55 °C; PEF with electric field intensity at 25–35 kV cm^−1^ and treatment time = 5–27 μs; and TT at T = 72 °C. The focus was on the processing conditions needed for each technology to give a 5-log microbial reduction from a starting inoculum level of roughly 10^6^ CFUmL^−1^. HPP gave 5.75-log reduction with 300 MPa|t = 1 min; HPU (thermosonication) gave a 5.69-log reduction with 55 °C, t = 3 min; PEF gave a 5.53-log reduction with 35 kV cm^−1^, t = 27 μs, 350 mL/min flow rate, 2 μs pulse width in monopolar mode; and TT contained no detectable *E. coli*. The authors concluded that the above non-thermal parameters had crucial roles in the microbial reduction ability, which was equivalent to thermal pasteurization.

The two technologies (HPP and HPU) were compared and tomato juice was treated with both to see which process had a better effect on the stability and in vitro bioaccessibility of carotenoids. Tomato juice was subjected to different HPP treatments at 200, 300, 400, and 500 MPa. HPU samples were treated with 200, 400, 600, and 800 W, with a constant frequency of 25 Hz. With the increase of pressure, the contents of all-*trans* lycopene decreased, while *cis*-lycopene isomers increased. The best HPP treatment was achieved at 200 MP, with a total lycopene content of 776.94 μg/100 g. The best HPU treatment was at 400 W, with a total lycopene content of 920.52 μg/100 g. The β-carotene and ζ-carotene contents were higher in HPP and HPU than in untreated samples, while HPP and HPU showed slight increases in the bioaccessibility of carotenoids. The highest lycopene bioaccessibility in HPP samples was obtained for all-*trans* (6.86%) and isomers (13.35%) at 500 MPa. In HPU samples, bioaccessibility peaks for all-*trans* (9.71%) and of isomers (15.82%) were achieved at 800 W. Afterwards, the HPP lycopene was fully liberated from the protein bonds and well dispersed in the tomato juice, making it fully available. On the other hand, ultrasound treatment caused less damage to the cell membranes, which resulted in lower pigment release. Therefore, it was concluded that HPP increases the antioxidant activity, while HPU slightly decreases it [106], which was attributed to their technological differences.

### 3.4. Hurdle Technologies Applied to Fruit Juices

In fruit juice manufacturing, important hurdle methods can be combined, which include: (i) application of low or high temperature; (ii) manipulation with water activity; (iii) acidity; (iv) redox potential; (v) application of chemical or natural preservatives; and (vi) application of competitive microorganisms (e.g., *Lactobacillus* species). These are valid methods for extending the shelf life and retaining the quality of a juice. The specificity of the hurdle concept is reflected in the synergistic effect of various mechanisms for inhibition or inactivation of target microorganisms [37]. Therefore, the innovative “obstacle” technology approach is an advanced approach to fruit juice processing with the potential to meet the high demands of consumers and manufacturers [107]. With the development of innovative process technologies, various novel hurdles have been investigated. Here, the technologies are applied in a certain sequence so that each of the non-invasive (“milder”) processing conditions gives the best results in terms of extending the shelf life and sensory quality [35,36]. Table 4 presents selected examples of the application of the hurdle approach for the preservation of functional fruit juices and beverages over the last 5 years.

One of the main problems with the production of fruit juices is browning. For instance, to avoid negative changes of color, apple juice was treated with HPP with the addition of α- and β-cyclodextrins (CD). CDs are allowed as additives in the food industry as natural antibrowning agents in fruit and vegetables juices [108]. The α- and β-CDs are officially approved as food additives in the EU, USA, and Japan, and α-CD is classified as a novel food by the EU [109,110,111]. For β-CD (E459), an acceptable daily intake of 5 mg kg^−1^ body weight per day has been established.

The concentrations of α- and β-CDs that were added to the juice were 10, 30, and 40 mM; and 5, 10, and 15 mM, respectively. The juice samples were exposed to HPP and browning was measured with the CIELab method. The control sample was untreated apple juice, while the rest of the samples were treated with HPP at 300, 400, and 500 MPa for 5 min at T = 22 °C. The browning index was calculated from L*, a*, and b* parameters, and phenolic compounds were measured using UHPLC. The results showed that HPP treatment did not cause color changes. Phenolic levels were higher in HPP samples, as increased pressure fostered extraction process. The majority of samples that contained 30 mM of α-CD had the least browning, while samples with 10 mM of α-CD showed the same effect but at 400–500 MPa. It was concluded that browning enzymes degraded with exposure to HPP and with the addition of α-CD, with the best results reached at 500 MPa exposure and 30 mM α-CD. For β-CD, the best results were observed with 15 mM, as higher concentrations were not tested due to limited water solubility. It was recommended that the use of β-CD can be accompanied by processing at 500 MPa [30].

Some studies have shown limited inactivation of bacterial endospores under PEF treatment; however, when combined with thermal energy, successful inactivation could be achieved [121]. Inactivation of *Bacillus subtilis* spores were studied in Ringer solution at different pH values (4 and 7), sugar levels (5% and 10%), and conductivity levels (4 and 15 mScm^−1^), as well as under various PEF operating conditions regarding the specific energy (up to 350 kJ/kg), electric field strength (6–11 kV/cm), and inlet temperature (56, 70, and 80 °C). The results revealed that the higher inlet temperature (80 °C) at 9 kV/cm and 10% of sugar content, which increased the electrical conductivity of the medium, was sufficient for successful microbial inactivation (3-log reduction) [121]. Additionally, it was found that *Bacillus subtilis* spores are more heat-sensitive in acidic environments [122].

The combined application of various technologies was further researched in blueberry juice [87]. The authors employed heat treatment (HT), sonication (SC), thermosonication (TS), manosonication (MS), manothermal (MT), and manothermosonication (MTS) to evaluate enzyme inactivation (PPO) and anthocyanin stability, as well as the reduction of *E. coli* O157:H7. Blueberry juice was treated with different temperatures (30, 40, 50, 60, 70, and 80 °C) and power settings (280, 420, 560, and 700 W) for 10 min. Afterwards, samples were treated with HT (80 °C), TS (40 °C/560 W), MT (350 MPa/40 °C), MS (560 W/5 min/350 MPa), or MTS (560 W/5 min/40 °C/350 MPa, 40 °C) for 5, 10, 15, and 20 min. Firstly, HT inactivated PPO (−2% after 5 min) and *E. coli* well, with 80% retention of anthocyanins. TS exposure slightly decreased the *E. coli* load by 0.17 log, with 23% PPO activity and 99% preservation of anthocyanins after the same length of time as for HT. MTS preserved 98% of anthocyanins and showed a 5.85-log reduction, with 11% PPO activity. Hence, combined treatment with sonication, high pressure, and mild thermal treatments has potential for the production of safe and nutritious blueberry juice [30].

The combined effects of ultrasound and PEF on quality of grapefruit juice were studied [123]. Grape juice was firstly treated with PEF (1 kHz, 20 kV cm^−1^, 600 μs, T < 45 °C), and subsequently the juice sample was sonicated using an ultrasound bath cleaner (28 kHz, 420 W, 20 °C, 30 min). The obtained results revealed that the acidity, soluble solids content, pH, electrical conductivity, and color parameters after treatment were not significantly changed in comparison to the control (untreated sample). As this hurdle concept resulted in decreased viscosity and increased cloud values as compared to control samples, the authors concluded that combined treatment with PEF and ultrasound might be used for the processing of grapefruit juice.

A recent study combined the use of HHP and ultrasound at 75 °C to achieve inactivation of *Aliclyclobacillus acidoterrestris* spores in apple juice. The juice was pretreated at 600 MPa for 15 min to weaken the spores. After the pressure treatment, the partially damaged spores were exposed to power ultrasound at 24 kHz and 20.2 WmL^−1^ for 60 min to reach the final spore reduction of 4.2 log. Nevertheless, the HPP-75 °C treatment was found to be the most effective for inactivating spores, resulting in 3.3-log reductions after 10 min vs. no inactivation for thermosonication (TS) and thermal processing [124].

The effects of combined treatment with US and HPP on enzymes (polyphenolase, POD, and PME), physicochemical properties, bioactive compounds, antioxidant activity, and microorganisms in apple juice (*M. domestica* cv. Fuji) were evaluated [125]. The combined treatment started with US (25 kHz and 70% amplitude) at 20 °C for 60 min, with subsequent HPP treatment at 250, 350, and 450 MPa for 10 min at room temperature. The obtained results indicated that the combined US-HPP treatment at 450 MPa caused the highest inactivation of enzymes, with complete inactivation of total plate counts, yeasts, and molds. The synergistic impacts of sonication and HHP also significantly improved the contents of phenolic compounds, ascorbic acid, antioxidant capacity, and color properties.

A combination of different hurdles, such as mild heat (54 °C for 10 min) or PEF treatments (25 pulses; 25 kV cm^−1^, 3.35 kJ cm^−1^ per pulse), and the addition of essential oil components (carvacrol, citral, and (+)-limonene) were employed to reduce spoilage in a clear apple juice [126]. For this purpose, PEF and the mild heat resistance of five strains of *Leuconostoc* spp. and five *Saccharomyces* spp. were tested, with different inactivation levels achieved for each treatment and strain. The authors concluded that combined processes could be useful alternatives for prolonging the shelf life of food products after moderate-intensity heat treatment, which would diminish the undesirable effects of high temperatures on food quality.

*Alicyclobacillus acidoterrestris* is a bacterium that is very resilient to high temperatures, as it is a thermoacidophilic, spore-forming, non-pathogenic microbe that grows at pH = 2.5–6.0 and temperatures ranging from 25 to 70 °C [67]. Acidic juices, such as apple juice, are a good medium for this microbe, which is able to cause spoilage. To that end, this study documented the exposure of apple juice to HPU and UV radiation. The juice was treated under UV-C radiation at 254 nm and 13.44 Wm^−2^, while HPU was used at 35 kHz frequency and 120–480 W power levels. Different combinations of conditions were used, namely: (i) sole UV-C; (ii) sole HPU; (iii) exposure of 25 min of HPU and 5 min of UV-C radiation; (iv) exposure of 5 min of HPU and 25 min of UV-C; (v) exposure of 5 min of UV-C and 25 min of HPU; (vi) exposure to TT at 95 °C (control); and (vii) exposure of TS in a thermostatic water bath at 95 °C. Spore inactivation was higher when ultrasound and thermal treatment were applied in combination than when each procedure was used separately. The use of UV-C alone severely reduced the number of spores by 5 logs in a short period of time. For the above parameters, the combined treatment (i) showed around a 5-log reduction; (ii) showed a low 1-log reduction; (iii) showed a 2-log reduction; (iv) showed a 5-log reduction; (v) showed a 4-log reduction; (vi) showed a 4-log reduction; and (vii) showed a 5-log reduction. HPU treatment alone had the lowest ability to reduce the microbial count, while thermal treatment (control) was comparable to sole use of UV-C, sole use of thermosonication, and combined treatment with 5 min of HPU and 25 min of UV-C. It was concluded that the results of this study provided new options for assuring food safety and quality in food manufacturing.

In 2015, a group of Mexican and Spanish scientists used combined alterations in pH, preservatives, and PEF treatment to inactivate *Saccharomyces cerevisiae* and *E. coli* in prickly pear juices. They found that *S. cerevisiae* was more resilient to pH reduction and addition of preservatives than *E. coli*, so the PEF was mainly used to inactivate *S. cerevisiae*. Interestingly, here pH was not just used to inactivate microorganisms, but also to increase their sensitivity to PEF treatment. The highest reduction of activity was accomplished with treatment parameters of 15 ls, 50 Hz, 36 kV cm^−1^ [127].

To test how technology modifies bioaccessibility, researchers mixed 75% of blended fruit juice (composed of orange, kiwi, pineapple, and mango) with 17.5% of water, milk, or soy milk and 7.5% of sugar, then the mixture was treated with PEF, HPP, or TT to evaluate the influence of carotenoids and antioxidant activity on bioaccessibility. PEF conditions were 35 kV cm^−1^ with 4 µs bipolar pulses at 200 Hz for 1800 µs, HPP conditions were 400 MPa at 40 °C for 5 min, while TT was performed at 90 °C t = 1 min. After the treatments, the bioaccessibility of all carotenoids was reduced in the samples, except in the case of *cis*-violaxanthin and neoxanthin, which increased by 79% in PEF and HPP samples. Losses of carotenoids were the highest during the TT, while samples with milk had the best results for bioaccessibility.

## 4. Conclusions

Juices are very important food commodities in the global market. Their preservation needs to account for microbial safety and thermally sensitive biologically active compounds. Pasteurization is a thermal treatment that has assured microbial safety in the past, but not without losses of the nutritive value of the juices. The hurdle technology approach, involving the combination of various preservation factors, has the potential to satisfy both aspects and to safely preserve the natural value of foods over extended periods of time.

Functional juices can be engineered from a number of biologically active ingredients, such as polyphenols, which have known antioxidative activity; or probiotic lactic acid bacteria, which need to be processed so that their positive aspects are retained in the final product.

Current markets demand juices with native nutritive value, sensory appeal, and an extended shelf life, but without additives. It is difficult to preserve products in such a way with the usual thermal treatments (e.g., pasteurization), as quite often higher temperatures have detrimental influences on the nutritive value of foods. Hence, there is an ongoing need to combine alternative non-thermal technologies for juice preservation. The pulsed electric field approach is an electrotechnology and a good option for the preservation of liquid foods that is able to ensure microbial safety and preserve the nutritive value of foods. However, it might need to be combined with other methods to achieve full microbial inactivation. Ultrasound-based technologies are valid options for microbial inactivation in juices, however as with PEF, sole application of such technology is not sufficient to achieve inactivation of microbes.

To reach the full potential of this technology, it is often combined with short thermal treatments to achieve complete product safety. Although it is generally good for preserving the nutritive and sensory attributes of foods, ultrasound still can generate “off flavors” in juices, undesired changes in viscosity, formation of free radicals, and lipid degradation [42,128]. High-pressure processing is one of the best options for the preservation of juices. It causes microbial inactivation with selective preservation of probiotic bacteria, improves the bioaccessibility of important nutrients, preserves and improves the native quality of products, and provides enzymatic inactivation. However, microbial spores are quite resilient to pressurization, so this treatment is often supplemented with thermal treatments to achieve the full effect. UV radiation shows good inactivation ability, hence it could be a good addition to hurdle technologies.

Although there have been numerous attempts to identify the ideal combination of technologies and their parameters in the processing of juices, there is still a gap in the research in terms of combining two of the most promising hurdle technology techniques, such as high-pressure processing and pulsed electric field techniques. This is somewhat unexpected, as both of these technologies are sustainable, selective, and thermally sensitive—processing factors that are currently cherished in food manufacturing. Some shortcomings that need to be overcome with these technologies include improvements in microbial inactivation, larger numbers of tests at the industrial scale, lowering implementation costs, and providing more funding to the scientific community for experimentation with innovative industry solutions.

## Figures and Tables

**Table 1:** The application of pulsed electric field (PEF) treatment for the preservation of functional fruit juices and beverages over the last 5 years.

Juice Type	PEF Conditions	Nutritive/Physicochemical Quality	Microbial Safety	Key Conclusions	Reference
Mango (*Mangifera indica* Linnaeus) and papaya (*Carica papaya* Linnaeus) juices with added stevia infusion	Square-wave bipolar pulses, with pulse width of 2.5 μs. 20–40 kV cm^−1^ 100–360 μs stevia leaf infusion: 0–2.5% (w/v)	-ascorbic acid -total anthocyanins (TA) -total carotenoids (TC) -steviol glycosides -total soluble solid content -CIELab -hydroxymethylfurfural content (HMF)	/	-higher electric field strengths revealed higher ascorbic acid reduction. -higher electric field strengths resulted in stevia beverages with higher TA and TC contents. -the ratio between rebaudioside A and stevioside increased after PEF treatments. -higher electric fields led to significantly higher HMF value. -HMF and color variations were greater in beverages without stevia. -optimum PEF conditions with respect to bioactive compounds: 21 kV cm^−1^ during 360 μs with 2.5% stevia	Carbonell-Capella et al. (2016) [51]
Sour cherry juice Apricot and peach nectars	Square-wave bipolar pulses with 3 μs duration and 20 μs delaying timeFlow rate: 50 mL min^−1^ 24 kV cm^−1^ 125 Hz (66 μs, 8.4 Js^−1^) 250 Hz (131 μs, 16.8 Js^−1^) 400 Hz (210 μs, 26.9 Js^−1^)	-titratable acidity (TA) -electrical conductivity (EC) -Commission Internationale de l’Éclairage LAB (CIELab) -non-enzymatic browning index (BI) -total ascorbic acid content (TAAC) -total β-carotene content (TBC) -total monomeric anthocyanin content (TMAC) -aroma compounds -sensory analysis	/	-PEF treatment did not change 94% of the sensory properties and 64% (sour cherry juice), 60% (apricot nectar), and 30% (peach nectar) of the physical properties. -aroma compounds were affected by. -in all investigated samples. PEF treatment significantly changed 57% of a total of 73 identified aroma compounds. -PEF could be applied with different treatment times for the pasteurization of all investigated samples.	Evrendilek (2016) [52]
Apple juice (unclarified)	Cyclic PEF treatment—each cycle consisted of 50 pulses (one pulse every 30 s). Design of experiments (DOE): −30 kV cm^−1^ -number of cycles: 4, 6, 8 (total of 200, 300, and 400 pulses, respectively) -storage: 24, 48, and 72 h under refrigeration. T < 35 °C	-total vitamin C content -total polyphenols -antioxidant activity (ABTS)	-Mesophilic and psychrophilic actinomycetes -Microscopic fungi -Yeasts -Enterococci -*Salmonella* -*Staphylococcus aureus*	-Regardless of the number of pulses, PEF did not affect the contents of vitamin C or total polyphenols during storage. -PEF treatment and the number of pulses influenced antioxidant activity, which decreased immediately after the treatment and after 24 h of storage. -PEF treatment successfully inactivated food spoilage microorganisms. -increased number of pulses positively affected the reduction in number of studied microorganisms.	Dziadek et al. (2019) [53]
Pinot noir juices (*Vitis vinifera* L.) obtained at different maceration times (0, 2, 4, 8, and 14 days) after PEF treatments	PEF operating variables: -constant pulse width 20 µs -50 Hz -1.5 kV cm^−1^ -243 pulses (“PEF Low”) -1033 pulses (“PEF High”) -estimated specific energy inputs were 14.48 ± 0.11 kJ/kg and 69.99 ± 0.52 kJ/kg for “PEF Low” and “PEF High”, respectively T < 25 ± 2 °C	-vitamin C -total phenolic content -malvidin-3-*O* -glucoside content -DPPH scavenging activity -simulated in vitro human gastrointestinal digestion -cell culture experiments using Caco-2 cell lines -biomarkers for general cellular health and integrity	/	-PEF treatment increased juice yield and preserved intense juice color. -PEF pretreatment of grapes improved the release of malvidin-3-*O*-glucoside for 224%. -PEF treatment resulted in higher total phenolic content (+61%), vitamin C (+19%), DPPH scavenging activity (+31%), bioprotective capacity (+25% for cell viability and +30% for LDH leakage).	Ying Leong et al. (2016) [54]
Date juice (variety Bou-Hattem)	High-intensity pulsed electric field (HIPEF) operating variables: -bipolar square-wave pulses of 4 µs -35 kV cm^−1^ -100 Hz for 1000 µs T < 35 °C Thermal treatment at 90 °C for 60 s in a tubularheat exchanger. All samples were stored in darkness for 5 weeks at 4 °C.	-total phenolic compounds -CIELab color measurement -HMF determination -turbidity evaluation -pH -soluble solids determination	/	-HIPEF treatment preserved the nutritive and physicochemical quality of date juices during storage in comparison to thermally treated and control (untreated) samples. -after HIPEF treatment, juices revealed higher amounts of total phenols, which were better preserved during storage than that untreated and thermally processed samples. -HIPEF did not alter the color parameters. -HIPEF treatment reduced HMF content of date juice after processing and during storage in comparison to thermally treated samples. -all investigated physicochemical properties were better-preserved after HIPEF in comparison to thermally processed and control (untreated) samples.	Mtaoua et al. (2016) [55]
Orange juice Watermelon juice Coconut water	All PEF processing conditions werestudied in a continuous-flow system. Moderate-intensity PEF: bipolar square-wave pulses of E = 0.9 and 2.7 kV cm^−1^ pulse width: τ = 15, 100 or 1000 μs High-intensity PEF (used in industrial applications): monopolar square-wave pulses of E = 10 or 20 kV cm^−1^ and pulse width τ = 2 μs	/	*Escherichia coli* *Listeria monocytogenes Lactobacillus plantarum* *Salmonella* Senftenberg *Saccharomyces cerevisiae*	-moderate-intensity PEF was shown to be very effective and easy to scale, and thus could be an alternative for pasteurization of fruit juices. -Optimal PEF conditions, which could match those of pasteurization: E = 2.7 kV cm^−1^ τ = 1000 μs. -moderate-field PEF can be used for treatment of both high-acid and low-acid products, in contrast to high-intensity PEF, which is only suitable for high-acid products. -moderate-intensity PEF demonstrated slight differences in the degree of inactivation between the different microbial species tested, while high-intensity PEF indicated greater differences between the microbial species.	Timmermans et al. (2019) [56]
Cloudy apple juice	Low-intensity PEF: 12.5 kV cm^−1^ Flow 27.6 L/h Energy input 76.4 kJ/L Frequency 62 Hz T_inlet_ 37.6 °C T_outlet_ 59.5 °C Thermal pasteurization (TP): 72 °C/15 s High-intensity PEF: 12.3 kV cm^−1^ Flow 24.5 L/hEnergy input 132.5 kJ/L Frequency 94 Hz T_inlet_ 37.3 °C T_outlet_ 72.8–73.8 °C Thermal pasteurization (TP): 85 °C/30 s Storage: 3 weeks at 4 °C	-color measurement -turbidity and cloud stability -particle size distribution -polyphenol oxidase (PPO) activity -peroxidase (POD) activity -pectin methylesterase (PME) activity -total soluble solids (TSS) -sugar profile -pH, titratable acidity (TA) -organic acid profile -vitamin C -sensory analysis -volatile compounds		-PEF-treated juices differed from the untreated juice, showing higher lightness (L*) and redness (a*). -PPO, POD, and PME activities were greatly reduced by high-intensity PEF. -vitamin C and cloud stability decreased during storage. -significant changes in pH, titratable acidity, organic acid, and sugar contents were not observed. -esters noticeably increased in juices after PEF treatments in comparison to TP treatment, where ester degradation reactions occurred together with the formation of off flavors. -increased contents of fructose and glucose and decreased contents of sucrose were observed during storage in all juices.	Wibowo et al. (2019) [57]
Beverages formulated with a blend of fruit juices (orange, kiwi, pineapple, and mango) and water (WB), milk (MB), or soy milk (SB)	-High-intensity pulsed electric field (HIPEF): 35 kV cm^−1^ 4 μs bipolar pulses at 200 Hz for 1800 μs T < 35 °C Thermal treatment (TT): 90 °C for 1 min)	-in vitro gastrointestinal digestion -individual carotenoids -lipophilic antioxidant activity (LAA) -bioaccessibility	/	-after HIPEF treatment, the contents of several carotenoids increased by between 8% and 28%. -HIPEF was found to be more effective than TT in preserving the concentrations and bioaccessibility of carotenoids and other lipophilic compounds in terms of antioxidant activity of investigated beverages. -the beverage with the highest bioaccessibility of total carotenoids was MB, followed by SB and WB. -milk matrix (MB) in combination with HIPEF improved the bioaccessibility of carotenoids by 15% as compared with the untreated samples. -HIPEF and TT decreased the bioaccessibility of carotenoids in WB. -food matrixes and food processing are able to modify the bioaccessibility of carotenoids.	Rodríguez-Roque et al. (2015) [48]
Beverages formulated with a blend of fruit juices (orange, kiwi, pineapple, and mango) and water (WB), milk (MB), or soy milk (SB)	-High-intensity pulsed electric field (HIPEF): 35 kV cm^−1^ 4 μs bipolar pulses at 200 Hz for 1800 μs T < 35 °C Thermal treatment (TT): 90 °C for 1 min)	-vitamin C -individual phenolic compounds -total phenolic content (TPC) -hydrophilic antioxidant activity (HAA) -bioaccessibility	/	-HIPEF reduced the content of vitamin C (8%–15%) as compared with untreated samples. -TT negatively affected the stability of vitamin C (losses of 31%) in comparison to untreated samples. -HIPEF did not alter the bioaccessibility of vitamin C in comparison with untreated samples. -significant decrease in the vitamin C bioaccessibility was noticed in TT samples. -HIPEF treatment provoked increased content of several phenolic compounds in MB and SB. -food matrix and processing could modify the bioaccessibility of bioactive compounds	Rodríguez-Roque et al. (2015) [47]
Clarified pomegranate juice (Hicaz cultivar)	pulse duration: 3 µs pulse delay time: 20 µs frequency: 500 pps controlled flow rate: 60 mL min^−1^ DOE: 0, 17, 23, 30 kV cm^−1^ 5, 15, 25, 35 °C Total treatment time was estimated at 108.4 µs, with applied energies of 37.5, 50.3, and 65.3 J, respectively.	-pH -CIELab, browning index (BI) -total antioxidant capacity (TAC)–DPPH -total phenolic content (TPC) -total monomeric anthocyanins (TMAC) -total ascorbic acid (TAAC) -sensory evaluation	*E. coli* O157:H7 (EDL 931 04054) *S. aureus* (95047)	-electric field strength was the most significant factor in terms of bacterial inactivation. -the inactivation of *S. aureus* and *E. coli* O157:H7 in PEF-treated samples reached up to 4.47 and 5.43 log CFU/mL, respectively. -the decreases in the mean initial TAC, TMAC, and TAAC with increased temperature, electric field strength, and energy were not significant. -the sensory properties of flavor, taste, aftertaste, and overall acceptance were not affected by PEF alone or PEF mild heat treatment.	Evrendilek (2017) [45]

**Table 2:** The application of ultrasound (US) treatment for the preservation of functional fruit juices and beverages over the last 5 years.

Juice Type	US Conditions	Nutritive/Physicochemical Quality	Microbial Safety	Key Conclusions	Reference
Strawberry juices (*Fragaria x ananassa* Duch. cv. Aromas) with prebiotic fiber (inulin and oligofructose)	Ultrasonic cleaning bath (TestLab, Argentina) −40 kHz −180 W Time: 0, 15, 30 min Preservation treatment included US + addition of geraniol. Storage: 14 days at 5 °C	-total phenolic content (TPC) -total flavonoids content (TFC) -total antioxidant capacity (TAC) -ascorbic acid -sensory evaluation	Mesophilic bacteria Yeasts Molds Inoculation with *E. coli* O157/H7 and *L. innocua*	-optimal preservation conditions: inulin: oligofructose = 5: 3, 0.225 μL/mL of geraniol and ultrasound time equal to 0. -the optimum preservation treatment was highly effective in reduction of native microflora and inhibition of inoculated pathogens in juices. -the optimum preservation treatment did not have a negative influence on bioactive compounds or antioxidant capacity. -the optimum preservation treatment promoted the stability of prebiotic fibers added into juices during storage.	Cassani et al. (2018) [72]
Cloudy apple juice (cv. Golden Delicious)	High-power ultrasound treatment (HPU) -ultrasound probe system (UP 100H, Hielscher Company, Teltow, Germany). 100 W, 30 kHz -probes with tip diameters of 10 and 20 mm -amplitudes: 40% and 80% -time: 3, 6, 9 min Storage: 7 days at 4 °C	-total phenols (TP) -total flavan-3-ols (TFL) -in vitro antioxidant capacity (DPPH, FRAP)	/	-in comparison to untreated samples, HPU treatment caused reductions in TP of 32.94% and TFL of 21.66%, while DPPH and FRAP values declined by 23.76% and 27.49%, respectively. -all HPU variables significantly affected phenolic stability and antioxidant capacity. -7 days of cold storage revealed the highest reduction of TP (89.21%), followed by TFL (82.80%), DPPH (79.51%), and FRAP (66.04%). -lower reductions of TFL (46.97%), DPPH (20.55%), and FRAP (24.16%) were observed during cold storage in untreated samples than in ultrasound treated.	Bursać Kovačević et al. (2019) [73]
Pear juice (*Pyrus bretschneideri* Read.)	-ultrasonic processor (VC 750, Sonics and Materials Inc., Newtown, CT, USA) 750 W, 20 kHz, 12.7 mm Amplitude: 70% US pasteurization at: 25, 45, and 65 °C (US25, US45, and US65) for 10 min. Conventional pasteurization: 65 °C/10 min (P1) 95 °C/2 min (P2)	-enzyme activity: POD, PPO, PME -ascorbic acid content -total phenols and flavonoids -soluble solids content, pH, acidity -total antioxidant capacity	-total plate count, yeast and mold	-significant reduction in residual activities of POD (43.2%), PPO (37.83%), and PME (40.22%) were observed in US45 juices. -the highest enzyme inactivation was found in US65 juices, which exhibited residual activities of POD, PME, and PPO of 4.3%, 3.25%, and 1.91%, respectively. -a complete inactivation of microbes was found in P95 and US65 treatments. -significant increase in the contents of ascorbic acid, total phenols, and flavonoids was detected in the US25 samples. -Ultrasound pasteurization at 65 °C for 10 min ensured the best retention of investigated bioactive compounds and enzymatic and microbial inactivation.	Saeeduddin et al. (2015) [74]
Clarified pomegranate juice	US generator (500 W, 20 kHz; Vibra-Cell 505, Sonics and Materials, Inc., Newtown, CT, USA) 500 W, 19 mm diameter probe, <35 °C Amplitude: 50%, 75%, 100% Time: 0, 6, 12, 18, 24, 30 min Pulse intervals of 5 s on and 5 s off.	-CIELab -total monomeric anthocyanins -total phenols -soluble solids content, pH	*E. coli* ATCC25922 *S. cerevisiae* ATCC 2366	-5-log reduction in *E. coli* ATCC 25,922 as a surrogate for *E.coli* O157:H7 and 1.36-log reduction of *S. cerevisiae* inoculated into pomegranate juice were achieved after US treatment (100%, 30 min). -the contents of anthocyanins were decreased upon US treatment (75% and 100%, t > 18 min) -total phenols did not change significantly during US treatment. -US technology showed the potential for enhancements in both the safety and quality of pomegranate juice.	Pala et al. (2015) [75]
Apple/grape juice blend (50:50)	Sonicator (JY96-IIN, Ningbo Scientz Biotechnology Co. Ltd. Ningbo, China), 25 kHz, 70% amplitude -Thermal treatment 100 °C/4 min (blanching) -high-temperature short-time (HTST) 72 °C/15 s -Ultra-sonication 5 and 10 min -Thermo-ultrasound 5 and 10 min/40 °C -Thermo-ultrasound 5 and 10 min/50 °C -Ultrasonic probe	-polyphenolic profile -total phenols -total flavonols -total flavonoids -DPPH-free radical scavenging activity -total antioxidant capacity -soluble solids content (SSC), viscosity, turbidity, pH, and acidity -CIELab	/	-ultrasonication (5 and 10 min) indicated significant increase in antioxidant activity and anthocyanin content of treated samples as compared to other treatments. -regarding individual bioactive compounds, significant differences were observed among all the treatments in this study. -insignificant influence of all the examined treatments on pH and titratable acidity (TA) was observed. -blanching, HTST, ultra-sonication, and thermo-ultra-sonication treatments had significant effects on viscosity, turbidity, SSC, and color parameters.	Aadil et al. (2020) [76]
Bayberry juice	Ultrasonic processor (600 W; BILON-600Y, Bilon Co., Ltd., Shanghai, China), 13 mm diameter probe tip, 20 kHz. -amplitudes of 20%, 40%, 60%, 80%, and 100%, with corresponding ultrasonic intensity levels of 90, 181, 271, 362, and 452 Wcm^−2^, respectively. -treatment times of 2, 4, 6, 8, and 10 min, with pulse durations of 5 s on and 5 s off. Thermal processing (TP) 90 °C/1 min	-soluble solids content (SSC), pH, titratable acidity -ascorbic acid -monomeric anthocyanins-antioxidant activity (DPPH) -hydroxymethylfurfural (HMF) -browning degree (BD) -CIELab and polymeric color (PC)-superoxide dismutase (SOD) activity	/	-US did not affect pH, SSC, TA, or yellowness (b*) as compared to untreated samples. -HMF, PC, BD, and L* values increased with higher ultrasonic intensity and prolonged treatment time. -in contrast to anthocyanins, US at lower intensity for a short time did not affect the stability of ascorbic acid. -with increasing US intensity and time, bioactive compounds decreased. -SOD activity increased (21–28%) with short (2–6 min) treatment time and then decreased with extension of ultrasound processing. -US treatments (<450 Wcm^−2^, 8 min) were optimal for preserving the quality of bayberry juice compared with TP treatment.	Cao et al. (2019) [77]
Grapefruit juice	Ultrasonic cleaner (SB-600 DTY, Ningbo Scientz Biotechnology Company Limited, Ningbo, China), 600 W 28 kHz, power radiation 70%, 20 °C, 30, 60, and 90 min	-total carotenoids -total lycopene -total anthocyanins -individual phenolic compounds -viscosity -sugars (sucrose, glucose, and fructose) -mineral elements	The microbiological analysis [78]	-significant increases in total carotenoids, lycopene, sugar contents, and phenolic compounds and decreases in viscosity and microbials were found in all the US samples as compared to control. -maximum improvement was observed in the US samples treated for 90 min. -complete microbial reduction was not achieved after US treatment.	Aadil et al. (2015) [79]
Strawberry juice	VC-750 US unit (Sonics and Materials, Inc., Newtown, CT, USA) 20 kHz, probe diameter 12.5 mm Sonication time: 5, 10, 15 min HTST: 72 °C/15 s Storage: 2 weeks at room temperature	-CIELab -ascorbic acid content (AAC) -antioxidant capacity (AOC) -total phenolic content (TPC)	*E. coli* O157:H7	-5-log reduction of *E. coli* O157:H7 was achieved by US treatment for 5 min (5.04 log CFU/mL), 10 min (5.36 log CFU/mL), and 15 min (6.08 log CFU/mL). -higher retentiona of color parameters were achieved during US as compared to HTST. -AAC decreased in all samples during storage, although US10 and US15 samples showed higher retention of AAC as compared to control samples. -US15 samples demonstrated the highest AOC and TPC during storage.	Yildiz et al. (2019) [80]
Blueberry juice (*Vaccinium corymbosum*)	Continuous ultra-sonication system (Model CPX 500, Cole Palmer Instruments, Vernon Hills, IL, USA) Probe diameter 10 mm, 20 kHz, 500 W Amplitude: 40%, 80%, 100% T < 25 °C	-total anthocyanin content (TAC) -total phenol content (TPC) -antioxidant activity (AA) -total soluble solids, pH, and titratable acidity -CIELab	-Aerobic plate count (APC) -Total coliforms (TC) -Yeasts and molds	-increased US intensity (amplitude) resulted in greater reductions of APC, TC, and yeast. -molds were not detected in the juice samples. -the highest log reduction in total aerobes (1.36 log CFU/mL) was achieved with high-intensity (100% amplitude) treatment. -US treatments did not affected TAC and color parameters of treated samples. -the TPC content of US treated samples significantly increased with flow rate and amplitude.	Mohideen et al. (2015) [81]
Peach juice	Ultrasonic tip (ECO-SONIC, QR1000 Model, Brazil) 1000 W, 20 kHz, 1.26 cm^2^ titanium tip793.65 Wcm^−2^ Sonication time: 0, 3, 6, 10, 15 min Storage: 21 days at 25 °C	-microstructure, physical properties, and stability (optical microstructure, particle size distribution, pulp sedimentation, serum cloudiness) -CIELab -rheological properties	/	-pulp sedimentation was highly reduced by the US treatment. -juice consistency and serum cloudiness (turbidity) showed an increase upon US treatment, followed by a decrease and then a new increase with respect to processing time. -US could be used to improve the physical properties of peach juice without significant color changes during storage.	Rojas et al. (2016) [82]

**Table 3:** Application of high-pressure processing (HPP) for the preservation of functional fruit juices and beverages over the last 5 years.

Juice Type	HPP Conditions	Nutritive/Physicochemical Quality	Microbial Safety	Key Conclusions	Reference
Cloudy apple juice	400 MPa at room temperature for 3 min (HPP1) 600 MPa at room temperature for 3 min (HPP2) Storage: 3 weeks at 4 °C	-color measurement -turbidity and cloud stability -particle size distribution -polyphenol oxidase (PPO), peroxidase (POD), and pectin methylesterase (PME) activity -total soluble solid (TSS) -sugar profile -pH, titratable acidity (TA)–organic acid profile -vitamin C -sensory analysis -volatile compounds	/	-CIELab parameters did not significantly differ among HPP1- and HPP2-treated juices. -HPP did not completely inactivate PPO and POD. -due to high residual PME activity (>90%), cloud stability decreased during storage in HPP juices. -significant changes in pH, titratableacidity, organic acid, and sugar content between HPP1 and HPP2 were not observed. -increased content of fructose and glucose and decreased content of sucrose were observed during storage.	Wibowo et al. (2019) [57]
Beverages formulated with a blend of fruit juices (orange, kiwi, pineapple, and mango) and water (WB), milk (MB), or soy milk (SB)	HPP: 400 MPa at 40 °C for 5 min Thermal treatment (TT): 90 °C for 1 min)	-in vitro gastrointestinal digestion -individual carotenoids -lipophilic antioxidant activity (LAA) -bioaccessibility	/	-HPP improved the contents of *cis*-violaxanthin, anteraxanthin, lutein, and zeaxanthin in MB (between 12% and 37%) compared with the control (untreated) samples. -lower amounts of total carotenoids were observed in TT beverages in comparison to HIPEF ones. -HIPEF was found to be more effective than TT in preserving the concentrations and bioaccessibility of carotenoids and other lipophilic compounds with antioxidant activity in the investigated beverages. -the beverage with the highest bioaccessibility of total carotenoids was MB, followed by SB and WB. -HPP increased the bioaccessibility of carotenoids in SB beverages by 10%. -HPP and TT decreased the bioaccessibility of carotenoids in WB. -food matrix and food processing are able to modify the bioaccessibility of carotenoids.	Rodríguez-Roque et al. (2015) [48]
Beverages formulated with a blend of fruit juices (orange, kiwi, pineapple and mango) and water (WB), milk (MB), or soy milk (SB)	HPP: 400 MPa at 40 °C for 5 min Thermal treatment (TT): 90 °C for 1 min)	-vitamin C -individual phenolic compounds -total phenolic content (TPC) -hydrophilic antioxidant activity (HAA) -bioaccessibility	/	-HPP did not alter the content of vitamin C in comparison with untreated samples, with the exception of SB, where a decrease of 10.5% was found. -TT negatively affected the stability of vitamin C (losses of 31%) in comparison to untreated samples. -HPP did not change the bioaccessibility of vitamin C in comparison with control samples, except for MB, which increased by 8%. -significant decrease in the vitamin C bioaccessibility was noticed in TT samples. -HIPEF treatment provoked increased contents of several phenolic compounds in MB and SB. -food matrix and processing could modify the bioaccessibility of bioactive compounds	Rodríguez-Roque et al. (2015) [47]
Pomegranate juice (*P. granatum* L. cv. Hicaznar)	HHP: 200, 300, 400 MPa 5 °C, 15 °C, 25 °C 5 and 10 min Thermal treatment (TT): 85 °C/10 min	-pH, titrable acidity, °Brix -CIELab -total phenolic content (TPC) -total monomeric anthocyanin concentration (TMAC) -antioxidant (free radical scavenging) activity (AA) (DPPH) -ascorbic acid-mannitol	Mesophilic bacteria Yeasts Molds	-HHP juices indicated no significant decreases in AA, TPC, and TMAC as compared to TT samples. -HHP juices treated for 5 min exhibited had ascorbic acid but decreased content with HPP at 10 min. -lower mannitol content was detected in HHP juices as compared to control. -optimal HPP treatments regarding microbial inactivation: 400 MPa/15 °C/5 min and 400 MPa/5 °C/10 min.	Subasi and Alpas (2017) [92]
Açaí juice (*Euterpe oleracea*)	HPP: 400, 450, 500, 600 MPa 20 °C 5 min Thermal treatment (TT): 85 °C/1 min	-anthocyanins -non-anthocyanin phenolic compounds -tocopherols -antioxidant capacity toward oxygen and nitrogen reactive species (ORAC, HOCl-scavenging capacity, H_2_O_2_-scavenging capacity, effect on the formation of nitroso compounds (NOC)	/	-HPP technology could be successfully used to produce high-quality açaí juice, with better retention of anthocyanins and increases in the content of non-anthocyanin phenolic compounds and antioxidant capacity as compared to the untreated and TT juices. -α-tocopherol, γ-tocopherol, and vitamin E activity were not changed upon HPP treatment compared to the control. -in comparison to TT juices, only HPP-500 MPa revealed a significant decrease in α-tocopherol. -HPP optimal conditions: 500 MPa/5 min/20 °C with respect to nutritive quality.	da Silveira et al. (2019) [93]
Cloudy apple juice (*Malus domestica* Borkh. cv. Gloster)	HPP: 200, 300, 600 MPa 5, 25, 45 °C 1, 5, and 15 min Darkness storage at 4 ± 2 °C for 2, 4, 6, 8, and 12 weeks.	-total soluble solids and pH -sugar content -vitamin C -polyphenol oxidase (PPO) and peroxidase (POD) activity -total and individual polyphenols	/	-HPP decreased residual activity for polyphenol oxidases (<1%) and peroxidases (33%). -POD was found to be more pressure-, temperature-, and time-resistant compared to PPO. -HPP significantly decreased gallic acid, all flavanols, and dichydrochalcones. -storage time significantly affected the stability of individual polyphenols. -HPP enhanced the color stability in cloudy apple juices due to the inhibition of enzymatic reactions during storage time; thus, it can be considered for preservation of apple products.	Marszałek et al. (2019) [94]
Concord grape juice	HPP: 319, 350, 425, 500, 531 MPa 35, 60, 120, 180, 205 s 5 °C	/	Inoculation (10^7^ CFU/mL): *E. coli* O157:H7 *S. enterica* *L. monocytogenes*	-the combined effects of high pressure (extrinsic factor), acid pH (3.39), and the phenolic compounds (intrinsic factors to Concord grape juice) may have had significant effects on achieving log reductions greater than 5-log for pathogens. -*E. coli* O157:H7 was found to be more resistant to HPP as compared to *S. enterica*, while *L. monocytogenes* did not show growth in any sample, either before or after HPP. -HPP at moderate pressure (400 MPa) and short time (2 min) was effective in reducing the pathogens tested in Concord grape juice.	Petrus (2020) [95]
Pitaya–pineapple (*Stenocereus* sp.–*Fragaria ananassa*) beverage	HPP: 400, 500, 600 MPa 2, 5, and 10 min 27, 29, and 31 °C Pressure come-up times (CUTs) were 2, 5, and 10 min.	A beverage composed of 55% pitaya pulp and 45% (w/w) pineapple pulp. -pH, total soluble solids (TSS), moisture content -vitamin C -total phenolic compounds (TPC) -betalains -pectin methyl esterase (PME)	/	-400 MPa/CUT treatment caused an increase of vitamin C (64%) compared with untreated beverages. -TPC decreased by 13%–48% at 400–600 MPa/CUT-6 min. -HPP did not alter the contents of betacyanin or betaxantin in beverages (near 100% retention). -the highest PME inactivation (23%) was achieved at 600 MPa/2–10 min. -the content of vitamin C increased from 5% (600 MPa-CUT) to 64% (400 MPa/CUT).	Sandate-Flores et al. (2017) [96]
Aloe vera–litchi mixed beverage	High-pressure thermal processing (HPTP) 400–600 MPa 30–60 °C 0–15 min Pressure come-up times (CUTs) were 87 s and 135 s Beverage formulations: aloe vera/litchi (v/v,%) Sample 1 (20:80) Sample 2 (25:75) Sample 3 (15:85) Sample 4 (0:100)	-sensory evaluation -pH, TSS, acidity -CIELab -ascorbic acid -total phenolic content -antioxidant capacity -enzyme activity (PME, PPO and POD)	-natural microbiota population	-the best beverage formulation was aloe vera/litchi (v/v,%) = 15:85. -HPTP minimally affected physicochemical properties of evaluated beverages. -the temperature applied during the HPTP had major impacts on ascorbic acid, phenolics, and antioxidants. -ascorbic acid in beverage samples was reduced by up to 40% after HPTP treatment. -PME was determined as the most resistant enzyme, with maximum inactivation achieved up to 53%. -the optimal HPTP conditions: 600 MPa/15 min/56 °C resulted in 49% inactivation of PME and 74% retention of ascorbic acid	Swami-Hulle et al. (2017) [97]
Keitt mango juice	Highly Hydrostatic Pressure (HHP) treatments: HHP1:200 MPa/15 min HHP2:400 MPa/15 min HHP3:600 MPa/15 min Thermal treatment (TT): 80 °C/30 min	-volatile compounds -sensory evaluation (QDA)	/	-total of 35 volatile compounds were detected in Keitt mango juice (hydrocarbons: 85.94%, alcohols: 5.07%, esters: 4.94%). -12 major aroma-active compounds characterize the typical flavor of Keitt mango juice. -all treatments (HHP and TT) could alter degrees of aroma loss compared to fresh juice. -ester contents were found to reduce after both TT and HHP. -results of QDA revealed that fresh mango juice was the most accepted, followed by HHP and TT juices.	Zhang et al. (2019) [98]

**Table 4:** The application of the hurdle approach for the preservation of functional fruit juices and beverages over the last 5 years.

Juice Type	Hurdle Approach	Nutritive/Physicochemical/ Microbial Quality	Key Conclusions	Reference
Kiwifruit juice (*Actinidia deliciosa* cv. Hayward)	Ultrasound: 40 kHz, 180 W Sonication time: 10 min (US 10) 30 min (US 30) T < 20 °C Pomegranate extract (PE) 180 µg/mL US10 + PE180 US30 + PE180 Refrigerated storage: 0, 2, and 7 days	-individual phenolic compounds -sensory evaluation -antioxidant activity (AA) -CIELab -total aerobic mesophilic bacteria(MES) -yeast and molds (YM)	-at 7th day of refrigerated storage, US treatments for 10 and 30 min showed significant reductions on yeasts and molds counts as compared to control samples (0.96 and 1.40 log reductions, respectively). -the addition of a second hurdle technology to the US treatments increased the effectiveness in terms of microbial inactivation, which means that shorter US treatment time could be applied to juice when combined with PE. -addition of PE to US-treated juice can improve the retention of AA of sonicated juices. -this hurdle technology showed a potential for use in fruit juice industry.	Tomadoni et al. (2015)
Strawberry juice (*Fragaria x ananassa* Duch, cv. Camarosa) enriched with fiber (oligofructose)	Ultrasound: 40 kHz, 180 W Sonication time: 0, 15, 30 min Vanillin: 0–1.25 mg/mL Juice formulation: inulin/oligofructose proportion = 1:3, 1:1, 3:1 Refrigerated storage: 14 days	-*Saccharomyces* spp. strains -total aerobic mesophilic bacteria (MES) -*Enterobacteriaceae* and total coliforms (EB) -yeasts and molds (YM) -sensory evaluation (QDA)	-microbiological indices were strongly affected by addition of vanillin regardless of the US time and fibers proportion were assayed. -vanillin and US resulted in critical factors for allsensory attributes studied. -fibers proportion did not modify microbiological or sensory indices. -Optimal hurdle approach conditions: 1.25 mg/mL of vanillin, 7.5 min of ultrasound time, and 5:3 ratio of inulin/oligofructose	Cassani et al. (2017) [112]
Orange and cloudy apple juices	Ultrasound–ultraviolet irradiation treatment + additives Dynashock multifrequency ultrasound waves: 600 W 28, 45, and 100 kHz at 1 ms time Heat treatment: 45, 50, 52, 55, or 60 °C UV-C lamp: 15 W Additives: sodium benzoate, potassium sorbate, α- and β-pinene	-*E. coli* O157:H7	-at all tested heating temperatures, *E. coli* O157:H7 was inactivated exponentially as the organisms were heated in the suspending medium. -5-log reduction pf *E. coli* O157:H7 could be achieved at 45, 50, 52, 55, and 60 °C, equivalent to 481.5, 103.6, 45.0, 22.4, and 10.54 min, respectively. -during US treatment, the temperature increased faster in the orange juice, resulting in a faster inactivation rate as compared to apple juice. -heat liberated by US cavitation resulted in 85% of the reduction in E. coli population. -the greater efficiency of the combined treatments (US+UV-C) was found in apple rather than in orange juice. -for cloudy and pigmented samples, the efficacy of the UV-C treatment was limited in terms of reducing the microorganisms on the surfaces of the treated samples. -combined US+UV-C treatment resulted in significantly faster microbial inactivation than singular US or UV-C treatments, especially in apple juice.	Gabriel (2015) [113]
Apple juice -commercial(CAJ) -freshly pressed (NAJ)	Continuous flow through pulsed light system (PLc, 0.73 Jcm^−2^, 155 mL min^−1^, EEO: 1.8 × 10^3^–4.1 × 10^3^ kW h/m^3^/order), alone or combined with ultrasound (US, 30 min, EEO: 4.4 × 10^5^–1.1 × 10^6^ kW h/m^3^/order) at ambient temperature. Cold storage (4 °C): 12 days	-*Escherichia coli* ATCC 35218 -*Salmonella* Enteritidis MA44 -*Saccharomyces cerevisiae* KE 162 -indigenous flora -color evolution-sensory shelf life and consumer sensory field studies	-single PL treatment did not cause differences between CAJ and NAJ, resulting in 1.8–4.2-log reductions. -obtained results revealed that combined treatment (US + PLc) caused 3.7–6.3-log reductions of tested microbes and positively affected the browning prevention during storage. -inhibitory synergistic effect between US and PLc was observed in postponed mold and yeast recovery for 7 days of cold storage. -processed NAJ was positively evaluated by a group of consumers, who emphasized its fresh natural apple taste.	Ferrairo et al. (2016) [114]
Prebiotic cranberry juice fortified with fructo-oligosaccharides (FOS)	The juice was subjected to HPP for 5 min (450 MPa) and to ultrasonic treatment for 5 min (18 kHz, 500 W, 600 and 1200 WL^−1^), followed by HPP for 5 min (450 MPa).	-pH, total soluble solids, instrumental color parameters -organic acids -anthocyanins -antioxidant activity -fructo-oligosaccharides (FOS)	-combined treatment (US + HPP) is viable process for the treatment of prebiotic juices. -the combination of ultrasound (1200 WL^−1^) followed by HPP increased the amounts of cyaniding, peonidin, and malvidine derivatives in the prebiotic juice, which corresponded to an increase in chroma and decrease in luminosity.	Gomes et al. (2017) [115]
Guava juice (*Psidium guajava* L.) Mango juice (*Mangifera indica* L.)	Nanoemulsions of *Mentha* *piperita* L. essential oil (n-MPEO) or suspensions of MPEO (s-MPEO) in combination with mild heat (MHT) (50, 52, 54 °C; 10 min), PEF (20, 25, 30 kV cm^−1^; 150 μs), and HHP (150, 200, 300 MPa; 15 min) treatments.	-*Escherichia coli* O157:H7	-MPEO was found to be more effective in guava than in mango juice. -s-MPEO and n-MPEO exhibited a synergistic effect in combination with MHT, PEF, and HHP against *E. coli* O157:H7. -MHT at 54 °C for 10 min caused a 2.7-log and 2.3-log reduction of *E. coli* O157:H7 in guava and mango juices, respectively. -when s-MPEO or n-MPEO was combined with MHT, PEF, or HHP, an average 5-log reduction of *E.coli* O157:H7 was achieved, which varied with the tested concentrations, treatment intensity, and the food matrix. -n-MPEO in combination with MHT, PEF, and HHP can be considered as promising methods to ensure microbial safety in fruit juices.	De Carvahlo et al. (2018) [116]
Apple juice	Ultrasound: 40 kHz, 700 W, 1, 2, 3, 4, and 5 min Fumaric acid (FA): 0%, 0.05%, 0.1%, and 0.15% (w/v)	Three strains each of: *E. coli* O157:H7 (ATCC 35150, ATCC 43889, and ATCC 43890) *S. Typhimurium* (ATCC, 19585, ATCC 43971, and DT 104) *L. monocytogenes* (ATCC 15313, ATCC, 19111, and ATCC, 19115) -color parameters, pH -non-enzymatic browning index -total phenolic content	-combined US + 0.15% FA treatment for 5 min achieved 5.67, 6.35, and 3.47 log reductions in *E. coli* O157:H7, *S. Typhimurium*, and *L. monocytogenes*, respectively, with the 1.55, 2.37, and 0.57 log CFU reductions attributed to the synergistic effect. -US + 0.15% FA treatment (5 min) did not affect the product quality. -obtained results suggest that simultaneous application of US and FA is a novel approach for ensuring the microbial safety of apple juice.	Park et al. (2019) [117]
Açai juice (*Euterpe* *oleracea*)	Combined ultrasound and ozone treatment. Ultrasound (US): 19 kHz 350 and 700 J mL^−1^ 5 min Processing temperature: 32 ± 1.2 °C Direct immersion of ozone gas for 5 or 10 min. Ozone concentration (O_3_): 1.50 ppm Processing temperature: 25 °C	-pH and titratable acidity -cloud value and non-enzymatic browning -viscosity -antioxidant activity (DPPH and ABTS) -total phenolic contents (TPC) -total anthocyanin content (TAC) -peroxidase (POD) and polyphenoloxidase (PPO) activity -total mesophilic bacteria and mold and yeast counts	-as ozone single treatment decreased the TPC and single US increased the TPC in açai juice samples, the combined processes showed no significant difference in TPC when compared to control (untreated) samples. -two isolated processes (O_3_, US) reduced TAC in the juice, therefore a high reduction of TAC in the açai juice was also observed after the combined treatments. -increase of the energy density (US) and the concentration of the ozone in combined treatment favored the reduction of the POD activity. -for the combined treatments, the PPO activity was higher than the treatments with single processes. -the combination of processes was shown to significantly reduce the contamination of açai juice.	Oliveira et al. (2018) [118]
Mixed Satsuma mandarin (*Citrus unshiu* Marc.) and Hallabong tangor (*Citrus kiyomi* x *Citrus ponkan*) juice (MH)	PEF combined with heat. Juice was heated to 55 °C and 70 °C prior to PEF treatment: PEF1-55 °C, 19 kV cm^−1^, 170 kJL^−1^, 24 μs, 166 kHZ PEF2-70 °C, 16 kV cm^−1^, 100 kJL^−1^, 30 μs, 115 kHZ PEF3-70 °C, 12 kV cm^−1^, 100 kJL^−1^, 30 μs, 320 kHZ	-total mesophilic aerobes, yeasts and molds, and coliforms counts -color -total soluble solid content and pH -ascorbic acid -antioxidant capacity	-H-PEF processing at 70 °C (16 kV cm^−1^, 100 kJL^−1^) preserved the physicochemical parameters and antioxidant capacity of MH juice -the same treatment influenced changes in juice color and browning degree, while demonstrating a strong inactivation effect on indigenous microorganisms (reduced the aerobe, yeast/mold, and coliform counts in MH juice by 3.9, 4.3, and 0.8 log CFUmL^−1^, respectively). -high electric field strength enhanced microbial inactivation, even at a relatively low level of specific energy.	Lee et al. (2018) [119]
Pineapple juice (*Ananas comosus*)	Combined pressure–thermal treatments: -600 MPa at 75, 85, and 95 °C for 0, 2, 5, 10, and 15 min -95 °C at 300, 450, and 600 MPa for 0, 2, 5, 10, and 15 min. Thermal treatment (TT): 75–95 °C for 0 to 60 min treatment times	-pH, acidity, and °Brix -ascorbic acid -kinetic models	-TT alone at 75 to 95 °C; the pineapple juice revealed loss in ascorbic acid content (2% to 5%), while significant loss was observed with increases in process temperature and treatment time, following First-order kinetics (loss up to 39%). -no significant difference in ascorbic acid content was observed between HPP (300–600 MPa at 30 °C) and control (untreated sample), irrespective of pressure holding times or pressure come-up time. -combined pressure–thermal treatment increased the ascorbic acid degradation rate and could be fitted by first-order fractional conversion.	Dhakal et al. (2018) [120]
